# 3-D finite element analysis of the effects of post location and loading location on stress distribution in root canals of the mandibular 1^st^ molar

**DOI:** 10.1590/1678-7757-2016-0406

**Published:** 2018-01-24

**Authors:** Hong Gi YOON, Hyun Keun OH, Dong-Yul LEE, Joo-Hee SHIN

**Affiliations:** 1 Seoul National University Dental Hospital, Department of Conservative Dentistry, Seoul, South Korea.; 2 Korea University, Graduate School of Clinical Dentistry, Department of Orthodontics, Seoul, South Korea.; 3 Korea University Guro Hospital, Department of Orthodontics, Seoul, South Korea.; 4 Korea University Guro Hospital, Department of Conservative Dentistry, Seoul, South Korea.

**Keywords:** Finite element analysis, Molar, Post, Stress

## Abstract

**Objective:**

The purpose of this study was to evaluate, by using finite element analysis, the influence of post location and occlusal loading location on the stress distribution pattern inside the root canals of the mandibular 1^st^ molar.

**Material and Methods:**

Three different 3-D models of the mandibular 1^st^ molar were established: no post (NP) – a model of endodontic and prosthodontic treatments; mesiobuccal post (MP) – a model of endodontic and prosthodontic treatments with a post in the mesiobuccal canal; and distal post (DP) – a model of endodontic and prosthodontic treatments with a post in the distal canal. A vertical force of 300 N, perpendicular to the occlusal plane, was applied to one of five 1 mm^2^ areas on the occlusal surface; mesial marginal ridge, distal marginal ridge, mesiobuccal cusp, distobuccal cusp, and central fossa. Finite element analysis was used to calculate the equivalent von Mises stresses on each root canal.

**Results:**

The DP model showed similar maximum stress values to the NP model, while the MP model showed markedly greater maximum stress values. The post procedure increased stress concentration inside the canals, although this was significantly affected by the site of the force.

**Conclusions:**

In the mandibular 1^st^ molar, the distal canal is the better place to insert the post than the mesiobuccal canal. However, if insertion into the mesiobuccal canal is unavoidable, there should be consideration on the occlusal contact, making central fossa and distal marginal ridge the main functioning areas.

## Introduction

The tooth is a complex structure mainly composed of pulp, dentin, cementum, and enamel, which is surrounded by periodontal tissues including the periodontal ligament and alveolar bone^19^. It deals with the most important function of grinding and chewing of foods, which subsequently results in force generation and transmission from the crown to the alveolar bone. The natural biomechanical balance of the tooth is well suited to this purpose, with the material properties of its aforementioned components functioning together to achieve this end. Therefore, the tooth must withstand the stress exerted during the chewing process; if it does not, it will fracture, especially in the root, resulting in permanent loss of function^12^.

It is commonly accepted that severely damaged tooth, whether by dental caries or fracture, must undergo the root canal therapy. When an extensive amount of coronal structure is lost, the post would be the first choice of treatment to prevent the core material from being detached^6^ ^,^ ^8^ ^,^ ^18^ ^,^ ^26^. It is in agreement that the post, if needed in the posterior region, should be inserted in the largest and straightest canal; namely, the distal canal in the mandibular molars, and the palatal canal in the maxillary molars^26^. Root canal treatment, which is intended to save the tooth, may adversely cause iatrogenic damage, particularly during canal preparation, canal filling, and post preparation^2^ ^,^ ^23^ ^,^ ^28^. It is obvious that endodontic treatment, whether with the post or not, changes the balance of the tooth structure, so the different phenomenon would happen as a force transmission system.

The stress distribution pattern within the root is the most important determinant in the fracture mechanism^12^. Concentrated stress may initiate and promote crack propagation along weakened surfaces^14^. However, it is not easy to directly measure the stress exerted on the root. Finite element analysis is a numerical method to investigate the stress and strain exerted on relevant structures, and in turn predict where a fracture might occur^30^. Many authors used finite element analysis to estimate the effect of posts on the stress distribution patterns, mostly in incisor and premolar teeth^1^ ^,^ ^3^ ^,^ ^4^ ^,^ ^15^ ^,^ ^16^ ^,^ ^21^ ^,^ ^24^ ^,^ ^27^. However, few authors studied the molars in the same regard, and none of those focused on the root canals of molars^6^ ^,^ ^17^. Therefore, this study was designed to evaluate the influence of post location and loading location on the stress distribution pattern inside root canals of the mandibular 1^st^ molar.

## Material and methods

### 3-D model of the mandibular 1st molar

**Figure 1:**
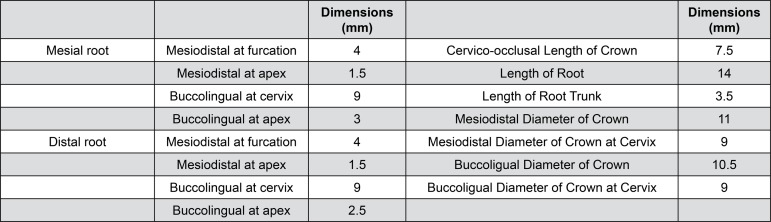
Dimensions of the mandibular 1st molar designed to this study for finite element analysis

A 3-D model of the mandibular 1^st^ molar having two roots was created by using Design Modeler™, a part of ANSYS™ v.15.0 software (ANSYS, Inc., Canonsburg, Pennsylvania, U.S.A.). External shapes of mesial and distal roots were generated based on the information obtained from the textbook, followed by the generation of five cusped coronal portion^20^. The canals, two on mesial and one oval shape on distal, were then fabricated inside the roots with average contour and curvature^5^ ^,^ ^10^ ^,^ ^11^. In the clinical view, the mesiobuccal canal and the mesiolingual canal had the same primary root canal curvature of 28 degrees, while the distal root canal had almost straight morphology showing slight curvature at its apical third in a distal direction^5^ ^,^ ^20^. In the proximal view, the primary root canal curvatures of mesiobuccal canal and mesiolingual canal were 21 degrees and 20 degrees each^5^. The canals were smoothly connected inside the coronal portion to form the pulp chamber in the quadrilateral cervical cross section, which is distally tapered from the wider buccolingual measurement of the mesial of the tooth. The roots were surrounded by 0.2 mm periodontal ligament, which was enclosed by supporting alveolar bone at an intact bone level 3 mm below the cementoenamel junction (CEJ). The tooth dimensions are shown in Figure 1 ^20^.

**Figure 2:**
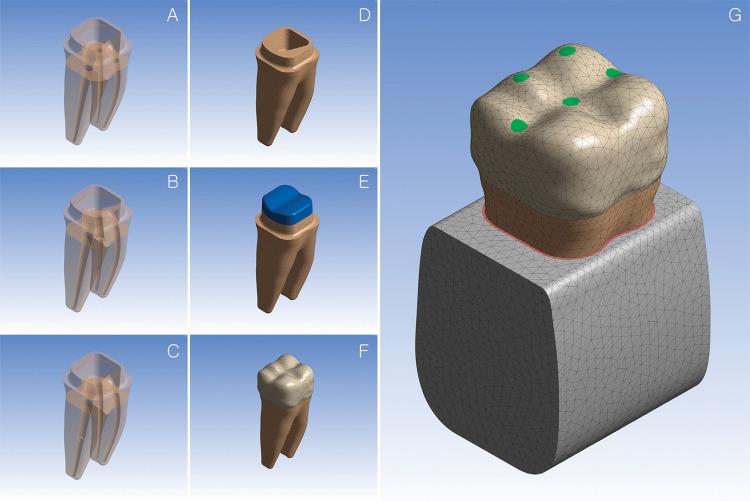
Transparent images of three different models showing internal root canals (A: no post model; B: mesiobuccal post model; C: distal post model). (D-G) Series of procedures to generate the mandibular 1st molar; (G) five loading locations are indicated on the crown

The model was subsequently subjected to access opening, root canal shaping and filling, mimicking the clinical procedures. All three canals were shaped with .06 taper instruments. After these procedures, the two mesial canals measured 1.3 mm in diameter at canal orifice, while the oval shape of distal canal had 2.0 mm in major axis and 1.3 mm in minor axis at canal orifice. The uppermost portions, at 2.0 mm from the canal orifices, of canal filling materials were then removed^26^. To insert the post, either the distal or mesiobuccal canal was subjected to drilling of ideal angulation and length. The drilling depths from the CEJ were 7.0 mm for the mesiobuccal canal and 8.0 mm for the distal canal. A post of 1.1 mm in diameter and 11 mm in length was inserted into the prepared bed of the distal or mesiobuccal root canal, with a 0.1 mm layer of cement around the post, and the empty space inside the teeth was filled with core material. To mimic a tooth with severe loss of coronal dentin, most of the coronal component was removed so that only 2.0 mm of the coronal portion above the cementoenamel junction was left; the removed portions were replaced with the restorative material. Finally, a 1.0 mm axial reduction and a 1.5 mm occlusal reduction were conducted, and the crown was added to the prepared coronal portion (Figure 2).

### Finite element analysis

**Figure 3:**
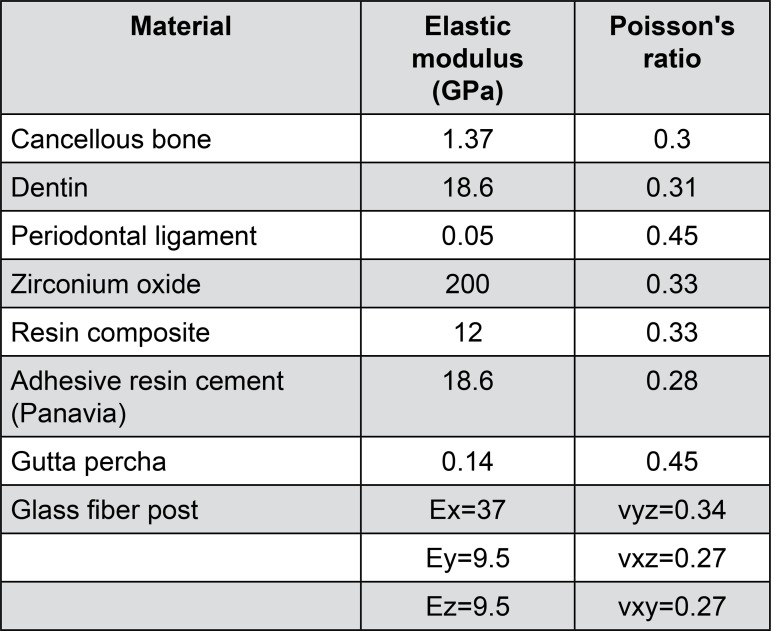
The elastic properties of the materials used for finite element analysis

The models were transferred to the static structural analysis system in ANSYS for finite element analysis. Zirconium oxide was chosen as the material for the prosthetic crown, and glass fiber for the post. The canal was filled using gutta-percha, and the post was cemented into the canal using Panavia. The core and coronal portion were constructed using resin-based composite material. All materials, other than the glass fiber post, were assumed to be homogeneous, isotropic, and linear elastic; the glass fiber post was considered as orthotropic, linear elastic material. The elastic moduli and Poisson’s ratio of the materials used in this study are shown in Figure 3 ^4^ ^,^ ^6^ ^,^ ^15^ ^,^ ^25^.

The 3-D meshes were generated using 10-node tetrahedral elements, with three degrees of freedom *per* node. The accuracy of the element size was checked so that same element size of 0.78 mm in average was achieved for each model to eliminate the mesh-dependent variation on the result. Thus, the meshing procedure generated about 200,000 nodes and 130,000 elements. All assemblies were assumed to be fully bonded, and the bottom of the alveolar bone was fixed to prevent any rigid dynamic motions. To assess the effect of tooth angulation, the long axis of the tooth was tilted 9 degrees mesially and 8 degrees lingually from the axis of occlusal loading^29^. A vertical force of 300 N, perpendicular to the occlusal plane, was applied to one of five 1 mm^2^ areas on the occlusal surface of the crown: the mesial marginal ridge, distal marginal ridge, mesiobuccal cusp, distobuccal cusp, and central fossa^7^ ^,^ ^9^.

## Results

**Figure 4:**
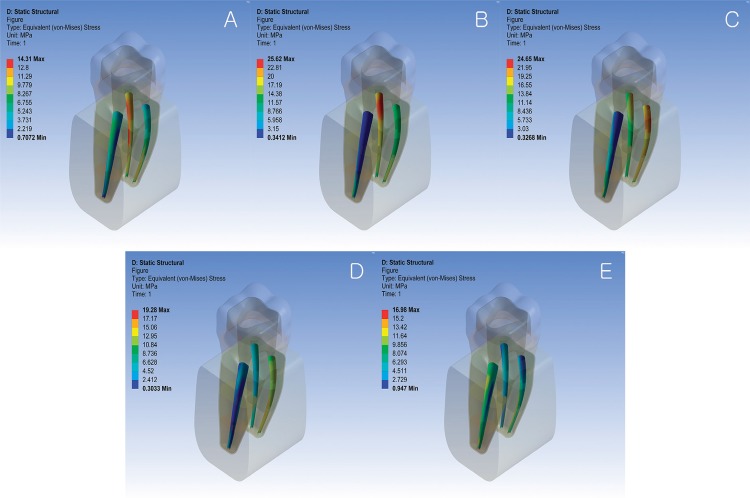
Equivalent von Mises stress distributions on the internal surfaces of root canals of the no post model under five different loading locations (A: distobuccal cusp; B: mesiobuccal cusp; C: mesial marginal ridge; D: central fossa; E: distal marginal ridge)

**Figure 5:**
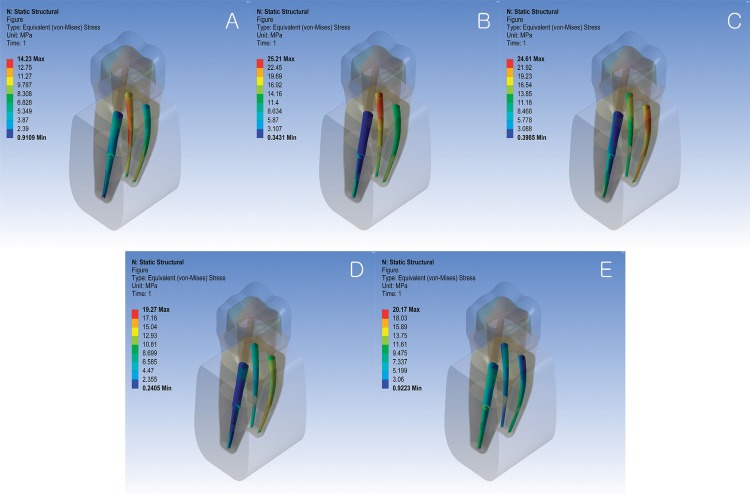
Equivalent von Mises stress distributions on the internal surfaces of root canals of the distal post model under five different loading locations (A; distobuccal cusp; B: mesiobuccal cusp; C: mesial marginal ridge; D: central fossa; E: distal marginal ridge)

**Figure 6:**
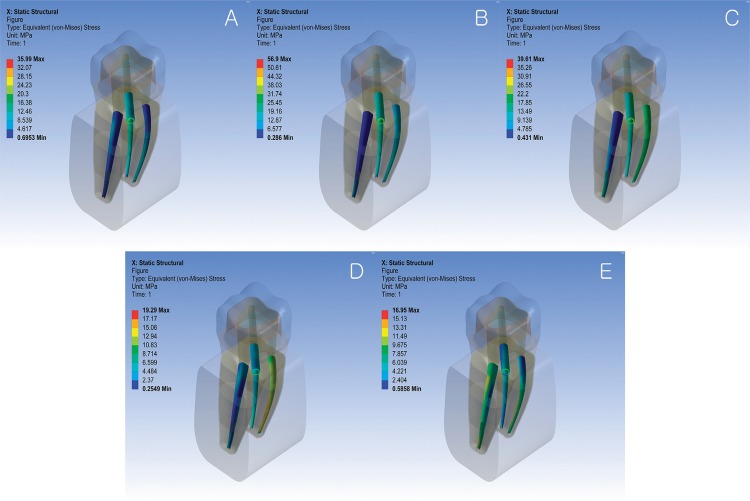
Equivalent von Mises stress distributions on the internal surfaces of root canals of the mesiobuccal post model under five different loading locations (A: distobuccal cusp; B: mesiobuccal cusp; C: mesial marginal ridge; D: central fossa; E: distal marginal ridge)

**Figure 7:**
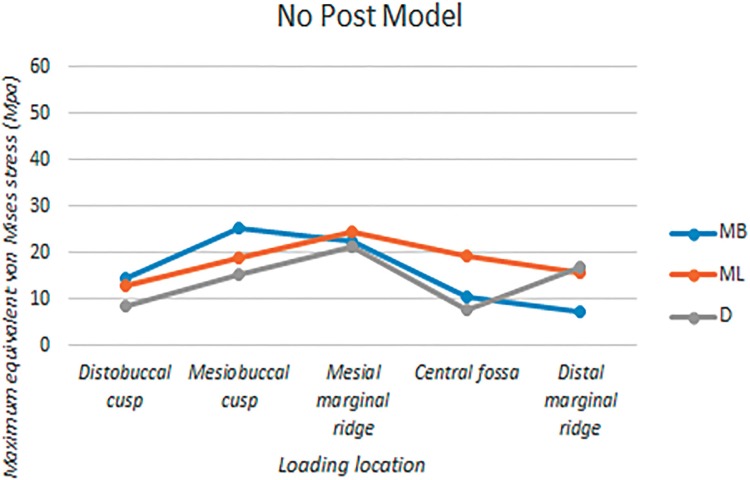
Maximum values of Equivalent Von Mises stresses on the inner surfaces of root canals of the no post model under five different loading locations. MB: mesiobuccal canal, ML: mesiolingual canal, D: distal canal

**Figure 8:**
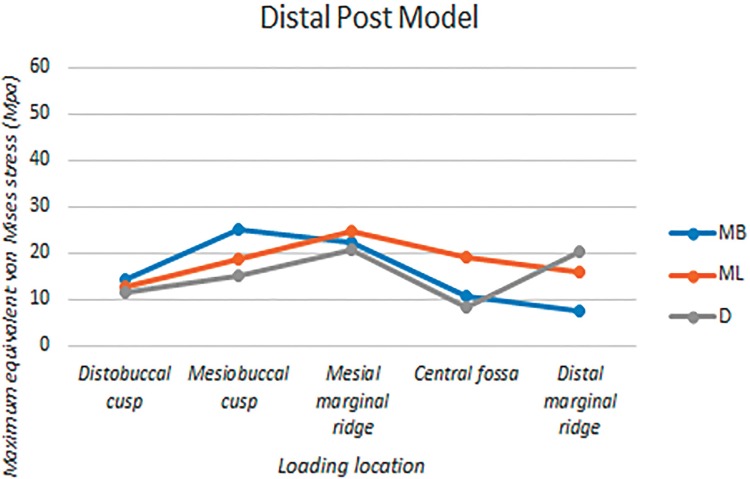
Maximum values of Equivalent Von Mises stresses on the inner surfaces of root canals of the distal post model under five different loading locations. MB: mesiobuccal canal, ML: mesiolingual canal, D: distal canal

**Figure 9:**
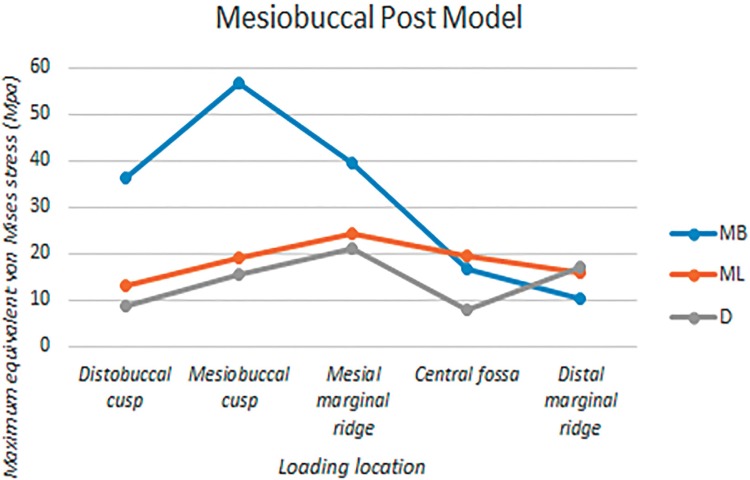
Maximum values of Equivalent Von Mises stresses on the inner surfaces of root canals of the mesiobuccal post model under five different loading locations. MB: mesiobuccal canal, ML: mesiolingual canal, D: distal canal

The root canals were divided into three portions: the coronal third, middle third, and apical third. Figures 4-6 show the equivalent von Mises stress distributions on the internal surfaces of root canals under five different loading locations. Table 1 shows the estimated maximum equivalent von Mises stress on the internal surface of each root canal at each portion. The largest among the three stress values from each canal is highlighted and plotted in Figures 7-9 to represent the canals in each model.

**Table 1:** Maximum values of Equivalent Von Mises stresses (MPa) on inner surfaces of root canals under five different loading locations (The largest among the three stress values from each canal is is highlighted)

Loading location		No post model			Distal post model			Mesiobuccal post model		
		Mesiobuccal canal	Mesiolingual canal	Distal canal	Mesiobuccal canal	Mesiolingual canal	Distal canal	Mesiobuccal canal	Mesiolingual canal	Distal canal
Distobuccal cusp	Coronal 1/3	13.59	6.72	8.59	13.48	6.6	5.15	13.28	6.43	8.52
	Middle 1/3	14.32	11.62	6.41	14.23	11.35	11.62	36	11.32	6.44
	Apical 1/3	14.04	12.83	5.2	14.05	12.81	5.21	13.98	12.83	5.2
Mesiobuccal cusp	Coronal 1/3	25.62	15.38	5.54	25.22	15.46	6.01	20.81	15.34	5.34
	Middle 1/3	22.41	15.78	6.4	22.38	15.53	10.52	56.9	15.53	6.34
	Apical 1/3	19.21	18.75	15.39	19.23	18.72	15	19.18	18.97	15.35
Mesial marginal ridge	Coronal 1/3	22.3	24.65	8.68	22.14	24.61	8.59	17.31	24.47	8.73
	Middle 1/3	16.57	23.19	8.83	16.54	23.26	15	39.62	22.98	8.87
	Apical 1/3	15.24	22.55	21.1	15.24	22.56	20.84	15.39	22.6	21.04
Central fossa	Coronal 1/3	9.52	15.32	6.7	9.3	15.39	5.29	6.52	15.17	6.8
	Middle 1/3	7.99	18.58	4.29	8.03	18.57	8.35	16.58	18.85	4.34
	Apical 1/3	10.64	19.28	7.85	10.63	19.27	7.95	10.92	19.29	7.97
Distal marginal ridge	Coronal 1/3	5.49	8.72	15.62	5.46	8.7	12.54	5.81	8.8	15.44
	Middle 1/3	5.91	15.25	12.5	5.91	15.71	20.17	10.08	15.68	12.48
	Apical 1/3	7.36	15.82	16.98	7.35	15.82	17.82	7.34	15.82	16.95

### NP Model

The locations in which the maximum stress values were determined in each canal varied as the area of force was changed. The mesial canals experienced more stress than the distal canal, except when the force was applied to distal marginal ridge. Most forces made the maximum stress occur within the apical third for the distal and mesiolingual canals. On the other hand, the location of maximum stress in the mesiobuccal canal depended on where the force was applied.

### DP Model

The two mesial canals showed stress distribution patterns similar to those of the NP model. In the distal canal, force applied to the distal marginal ridge, distobuccal cusp, and central fossa caused maximum stress in the middle third, where the apex of the post was located. However, when force was applied to the two mesial portions, the location of maximum stress in the distal canal was similar to that of the NP model the NP model, even though the stress distribution pattern was different.

### MP Model

The mesiolingual and distal canals showed stress distribution patterns similar to those in the NP model. Applying forces to the buccal or mesial portions of the crown significantly increased maximum stress values within the mesiobuccal canal, with the largest value occurring when loaded on the mesiobuccal cusp. Forces on the central fossa and distal marginal ridge changed the stress distribution patterns within the mesiobuccal canal, causing a slight increase in the maximum stress value. Regardless of the loading location, the maximum stress in the mesiobuccal canal occurred in the middle third.

## Discussion

The mandibular 1^st^ molar is the first permanent tooth to erupt, emerging at around six years old. For this reason, coronal loss because of dental caries or fracture is more likely in the mandibular 1^st^ molar, and such coronal loss may in turn require root canal treatment followed by post and crown procedure. The morphology of the mandibular 1^st^ molar does not vary as much as that of the mandibular 2^nd^ molar^20^. Moreover, the external and internal root shapes of the mandibular 1^st^ molar have been investigated and analyzed by many authors^5^ ^,^ ^10^ ^,^ ^11^ ^,^ ^20^. With such accumulated data, the “standard morphology” of the mandibular 1^st^ molar for finite element analysis was created.

Most authors in this field have designed their models by scanning a real tooth and adding the inner shape of the root canals to the outer shape obtained^4^ ^,^ ^6^ ^,^ ^15^ ^,^ ^16^ ^,^ ^24^ ^,^ ^27^. In this study, however, both outer and inner shapes of the tooth were designed using information accumulated from many previous authors^5^ ^,^ ^10^ ^,^ ^11^ ^,^ ^20^. In this way, it is possible to use the model to evaluate the influence of different root shapes, *i.e.*, different root lengths or curvatures, on the stress distribution pattern inside the root canals by simply altering the shape of root designed.

Procedures such as root canal preparation and filling, or post drilling, can cause damage, especially micro-cracks, on the inner surface of the root canals^2^ ^,^ ^23^ ^,^ ^28^. Furthermore, concentrated stress may initiate and promote crack propagation along these weakened surfaces^14^. However, no study has focused on the internal surfaces of root canals of molar teeth before our research. Dejak and Mlotkowski^6^ (2013) compared the equivalent stresses in molars restored with endocrowns as well as posts and cores. They found that the higher equivalent stresses occurred in molar restored with posts, but they mentioned no equivalent stresses inside the root canals. On the other hand, Lu, et al.^17^ (2013) analyzed the Von Mises stresses both at the root canal orifice and inside the root canal with different adhesives^17^. In their study, they reported stress concentration near the root canal and at the bottom of the root canal, irrespective of adhesive materials. However, they did not estimate the influence of the post on the stress distribution pattern inside root canals. Therefore, it was worth to numerically analyze the influence of the post insertion with variant loading locations on the stress distribution pattern and the stress concentration inside the root canals of the molar.

The diameter of the post drill was larger than that of the root canal, which resulted in the generation of internal and external line angles of dentin at the post apex (Figure 2). While the internal line angle could be rounded using a post drill with a round end, it was not possible to properly round off the external line angle; this caused the stress concentration on the middle third of post inserted canals. On the contrary, maximum stress was reduced in the coronal third under most of the five forces; it could therefore be said that post procedure moved stress from the coronal third to the middle third. This result is supported by the previous study of Cailleteau, et al.^3^ (1992), in which the maximum stresses were shifted to the apical end of the post by the post placement. Reinhardt, et al.^22^ (1983) also found the stress concentration in the small amount of dentin remaining near the post apex, which agrees with our result^22^. This result can also be supported by the study of Liu, et al.^16^ (2014), in which they reported the stress increase at the apex and the decrease in the coronal root.

The mesial and distal root canals display different stress transmission characteristics, mainly because of a difference in morphology and mesiolingual angulation of the tooth. In our study, the mesial canals transferred stress less proficiently and showed high stress concentration. It follows that there is a greater chance of crack generation and propagation in the mesial canals. In the distal canal of the NP model, maximum stress was mostly exerted on the apical third. This could be explained by the influence of the straightness of the distal canal and the decrease of the circumference of root canal as it goes to the apex. Also, the value of maximum stress was significantly affected by the loading locations, due to the forces in different locations generated at different moments on the distal canal. In the DP model, even though the post procedure increased the stress concentration in the middle third of the distal canal, the increments were not high enough to change the maximum stress pattern in the distal canal. This meant that the maximum stress pattern of the distal canal in the DP resembled that in the NP. On the other hand, the location of maximum stress varied in the mesiobuccal canal of the NP model, depending on where the force was applied. This would be explained by the fact that the curved mesiobuccal canal, would be in different moments and force environments with varying locations of the load. In the mesiobuccal canal of the MP model, the post procedure increased the stress concentration in the middle third to a much greater degree, especially when loaded on distobuccal cusp, mesiobuccal cusp, and mesial marginal ridge. This could be partially explained by the different distance from the axis of force to the apex of the post. Among the five loading locations, the force on mesiobuccal cusp, in which the distance from the axis of force to the apex of the post was the closest, made the largest maximum stress increment in the middle third of the mesiobuccal canal. This could be the explanation for the difference in maximum stress pattern on the mesiobuccal canal between the NP and the MP models.

Many authors have used finite element analysis to compare the effect of post material on stress distribution patterns in the roots; most have concluded that a glass fiber post with an elastic modulus similar to dentin results in a well-distributed stress pattern with minimal concentration of stress^1^ ^,^ ^15^ ^,^ ^16^ ^,^ ^21^ ^,^ ^24^ ^,^ ^27^. In our study, the glass fiber post was chosen because it is expected to induce a smaller stress concentration than the other post materials. The influence of post material, as well as post diameter and length, on the stress distribution pattern should be analyzed in future studies.

In this study, all materials, except the glass fiber post, were assumed to be homogenous, isotropic, and linearly elastic; however, this may not match clinical reality. The periodontal ligament is a vascular connective tissue composed principally of collagen fibers and tissue fluids^19^. This implies that the assumptions of homogeneity, isotropicity, and linear elasticity must have been erroneous. However, for simplicity, and utility on finite element analysis, many authors have capitulated to the assumption and focused on finding the most suitable elastic modulus values of periodontal ligament as a homogenous, isotropic, and linear elastic material^3^ ^,^ ^6^ ^,^ ^16^ ^,^ ^24^ ^,^ ^25^. However, recent authors have regarded the periodontal ligament as a non-linear, hyperelastic material, and have found variable elastic modulus values for periodontal ligament^13^. The influence of periodontal ligament as a non-linear hyperelastic material should be considered in further studies.

In this study, because of the curvature of the mesiobuccal canal, the post could not be positioned as deep as the half of the bone insertion. Instead, the mesiobuccal post bed was prepared to the half of the root, which was the 7 mm from the CEJ, so that as little dentin would be removed as possible. Similarly, the distal post was inserted into the 8 mm from the CEJ to get rid of the possible influence of post length on the results. Moreover, only the vertical force on five areas of the occlusal surface was considered. However, chewing food can generate force anywhere on the crown, and in any direction. Besides, the young are susceptible to dental caries, while periodontal disease and sequential bone loss are more common in the aged. Therefore, the influences of the quantitative surrounding bone level as well as the force in other directions, especially horizontal, should be further investigated.

## Conclusion

Within the limitations of our study, we concluded the following for the mandibular 1^st^ molar:

1. Stress distribution pattern was significantly influenced by force location.
2. The post procedure increased the stress concentration in the post-placed root canal, especially in the middle third.
3. The distal canal is a better place to insert the post than the mesiobuccal canal.
4. If insertion into the mesiobuccal canal is unavoidable, then the central fossa and distal marginal ridge should be the main functioning areas, avoiding buccal and mesial aspects on the crown from being loaded as possible.
